# Effects of enhanced cutaneous sensory input on interlimb strength transfer of the wrist extensors

**DOI:** 10.14814/phy2.14406

**Published:** 2020-03-27

**Authors:** Trevor S. Barss, Taryn Klarner, Yao Sun, Kristy Inouye, E. Paul Zehr

**Affiliations:** ^1^ Rehabilitation Neuroscience Laboratory University of Victoria Victoria BC Canada; ^2^ Human Discovery Science International Collaboration on Repair Discoveries (ICORD) Vancouver BC Canada; ^3^ Centre for Biomedical Research University of Victoria Victoria BC Canada; ^4^ School of Kinesiology Lakehead University Thunder Bay ON USA; ^5^ Division of Medical Sciences University of Victoria BC Canada; ^6^ Zanshin Consulting Inc. Victoria BC Canada

**Keywords:** Cross‐education, cutaneous, electrical stimulation, electromyography, plasticity, reflexes, resistance training

## Abstract

The relative contribution of cutaneous sensory feedback to interlimb strength transfer remains unexplored. Therefore, this study aimed to determine the relative contribution of cutaneous afferent pathways as a substrate for cross‐education by directly assessing how “enhanced” cutaneous stimulation alters ipsilateral and contralateral strength gains in the forearm. Twenty‐seven right‐handed participants were randomly assigned to 1‐of‐3 training groups and completed 6 sets of 8 repetitions 3x/week for 5 weeks. Voluntary training (TRAIN) included unilateral maximal voluntary contractions (MVCs) of the wrist extensors. Cutaneous stimulation (STIM), a sham training condition, included cutaneous stimulation (2x radiating threshold; 3sec; 50Hz) of the superficial radial (SR) nerve at the wrist. TRAIN + STIM training included MVCs of the wrist extensors with simultaneous SR stimulation. Two pre‐ and one posttraining session assessed the relative increase in force output during MVCs of isometric wrist extension, wrist flexion, and handgrip. Maximal voluntary muscle activation was simultaneously recorded from the flexor and extensor carpi radialis. Cutaneous reflex pathways were evaluated through stimulation of the SR nerve during graded ipsilateral contractions. Results indicate TRAIN increased force output compared with STIM in both trained (85.0 ± 6.2 Nm vs. 59.8 ± 6.1 Nm) and untrained wrist extensors (73.9 ± 3.5 Nm vs. 58.8 Nm). Providing ‘enhanced’ sensory input during training (TRAIN + STIM) also led to increases in strength in the trained limb compared with STIM (79.3 ± 6.3 Nm vs. 59.8 ± 6.1 Nm). However, in the untrained limb no difference occurred between TRAIN + STIM and STIM (63.0 ± 3.7 Nm vs. 58.8 Nm). This suggests when ‘enhanced’ input was provided independent of timing with active muscle contraction, interlimb strength transfer to the untrained wrist extensors was blocked. This indicates that the sensory volley may have interfered with the integration of appropriate sensorimotor cues required to facilitate an interlimb transfer, highlighting the importance of appropriately timed cutaneous feedback.

## Graphical abstract

No study has directly assessed the relative contribution of somatosensory feedback to interlimb strength transfer. Providing ‘enhanced’ cutaneous input during unilateral wrist extension training blocked the interlimb strength transfer to the untrained limb that was seen when participants trained with voluntary contractions only. This study highlights the importance of appropriate cutaneous sensory feedback to the interlimb transfer of strength following unilateral training.

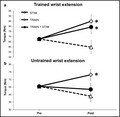

## 1 INTRODUCTION

Unilateral training for bilateral strength gains has recently been highlighted for its possible use as a rehabilitation strategy during recovery from asymmetrical injuries (Barss, Pearcey, & Zehr, 2016; Farthing & Zehr, 2014; Hendy, Spittle, & Kidgell, 2012). ‘Cross‐education’, ‘inter‐limb strength transfer’, or the ‘cross‐transfer’ effect is a neural adaptation defined as the increase in strength or functional performance of the untrained contralateral limb after unilateral training (Farthing & Chilibeck, 2003; Lee & Carroll, 2007; Ruddy & Carson, 2013; Scripture, Smith, & Brown, 1894). While its use as an adjunct therapy during rehabilitation from unilateral injury continues to be explored, a major focus of research has shifted into optimizing how unilateral training will be incorporated to maximize strength or performance gains. The idea of “enhanced” sensory integration during unilateral training has recently been explored through the use of mirror box therapy (Howatson, Zult, Farthing, Zijdewind, & Hortobágyi, 2013). It remains unknown if incorporating other sensory modalities (e.g., tactile somatosensory feedback) during resistance training may provide a similar enhancement effect.

While the original work on cross‐education by Edward Wheeler Scripture (Scripture et al., 1894) was published over a century ago, its origins stem from Alfred Wilhelm Volkmann (1801–1877) who found that performing unilateral sensory acuity training using a two‐point discrimination task, improved performance bilaterally (Volkmann, 1858). This work was not only the first to identify a portion of the phenomenon that would later be coined ‘cross‐education’ but also highlights the effect of using cutaneous afferent sensitivity training. It is well established that cutaneous sensory information can have widespread effects in sculpting motor output (Duysens, 1977; Panek, Bui, Wright, & Brownstone, 2014; Zehr & Stein, 1999) by providing accurate perceptual information about joint position and movement proprioception and kinesthesia (Collins & Prochazka, 1996; Collins, Refshauge, & Gandevia, 2000; Collins & KM R, Todd G, Gandevia S., 2005; Proske & Gandevia, 2012). This includes alterations in contralateral muscles with electrical stimulation provided unilaterally to cutaneous nerve branches (Haridas & Zehr, 2003; Zehr, Collins, & Chua, 2001). Previously, electrical stimulation provided to the radial and median mixed peripheral nerves has been shown to alter reciprocal inhibition in the contralateral upper limb. Interestingly, reciprocal inhibition in the contralateral limb was reduced by 16.5% with radial nerve stimulation. However, stimulation of cutaneous branches of each nerve did not alter reciprocal inhibition in the contralateral limb indicating that multiple converging pathways are important to understand interlimb interactions of electrical stimulation (Delwaide & Pepin, 1991). Therefore, one possibility is that providing ‘enhanced’ cutaneous input may interact with mechanisms and pathways responsible for cross‐education and alter the transfer of strength to the untrained limb.

Previously, adaptations in spinal reflex pathways have been shown to occur with unilateral training which may contribute to cross‐education. On the trained side, previous studies have shown increased H‐reflex amplitude (Lagerquist, Zehr, & Docherty, 2006), increased H‐reflex amplitude at threshold (Dragert & Zehr, 2011), and increased reciprocal inhibition (Geertsen, Lundbye‐Jensen, & Nielsen, 2008). On the untrained side, little evidence of change in H‐reflex amplitudes have been noted in the agonist muscle despite an increase in strength (Del Balso and Cafarelli, 2007; Fimland et al., 2009; Lagerquist, Zehr, & Docherty, 2006). However, maximal H‐reflex amplitude has been shown to be reduced in the antagonist muscle after unilateral plantar flexion training in a neurologically intact group, (Dragert & Zehr, 2011) while spinal reflex excitability and reciprocal inhibition within the untrained more affected tibialis anterior were altered in a poststroke population (Dragert & Zehr, 2013). In recent years, evidence of a cortical contribution to cross‐education has been established. Two nonexclusive theories have been proposed: the ‘cross‐activation’ and ‘bilateral access’ hypotheses (Aniss, Gandevia, & Burke, 1992; Négyesi et al., 2018; Russmann, Lamy, Shamim, Meunier, & Hallett, 2009). It is clear that the ‘untrained’ motor cortex, ipsilateral to the trained limb, plays a critical role in mediating the cross‐transfer effect (Russmann et al., 2009). As well, recent chronic voluntary strength studies using transcranial magnetic stimulation (TMS) and functional magnetic resonance imaging (fMRI) have confirmed that reduced interhemispheric inhibition and increased activation of specific areas in the nonexercised hemisphere are key moderators of cross‐education in healthy adults (Geertsen et al., 2008; Hortobágyi, Taylor, Petersen, Russell, & Gandevia, 2003).

Unfortunately, little to no work has explored the role of cutaneous sensory feedback at any level of the nervous system during resistance training. Understanding if cutaneous sensory information can impact strength gains in the trained or untrained limb will provide information toward a unifying model of cross‐education. It has previously been suggested that heightened afferent input associated with electrical muscular stimulation plays a key role in neural adaptations to electrically stimulated strength training (Hortobágyi & Maffiuletti, 2011). Providing electrical stimulation to the wrist extensor or flexor muscles has been shown to increase activation of the contralateral primary motor cortex, primary somatosensory cortex, premotor cortex, and numerous other areas important in motor control (Blickenstorfer et al., 2009) with bilateral activation of supplementary motor areas ( Han et al., 2003). Providing a large sensory volley during unilateral resistance training may interact with many of these same cortical areas that contribute to cross‐education.

The addition of ‘enhanced’ somatosensory input to facilitate the transfer of a motor skill has been proposed in an analytic review by Veldman, Maffiuletti, Hallett, Zijdewind, and Hortobágyi (2014) as prolonged low‐amplitude somatosensory electric stimulation (SES) with nerve stimulation can have ‘direct’ and ‘crossed’ effects on brain activation, corticospinal excitability, and motor performance which could enhance transfer effects of unilateral training. An initial study indicated that a single session of both unilateral SES applied to the median and radial nerves alone and performance of a visuomotor performance task at the wrist alone improved task performance in both the trained and untrained limbs (Veldman et al., 2015). However, the addition of SES during the visuomotor task did not enhance the transfer to a greater extent and it was concluded they may be mediated by different mechanisms. This is confirmed in a follow‐up study which highlighted that 20 min of low‐amplitude SES applied to the median and radial nerves alone facilitates interlimb transfer of visuomotor performance. However, a recent pilot study in healthy young adults found a single session of SES paired with a visuomotor task did not improve the transfer of practice‐induced skill transfer to the untrained limb of either SES or skill training alone (Négyesi et al., 2018).

Recently, the effect of a single session of unilateral strength training combined with transcranial direct‐current stimulation (tDCS) applied to the ipsilateral (untrained) M1 on strength was assessed (Hendy & Kidgell, 2014). Researchers found that strength of the untrained, left extensor carpi radialis (ECR) increased following training of the right ECR with tDCS of the right M1, but not following training of the right ECR with sham‐tDCS or tDCS alone. This was accompanied by neural modulation in the ipsilateral M1, including an increase in corticospinal excitability, a decrease in short latency intracortical inhibition (SICI), and an increase in cross‐activation during maximal contractions in the right ECR. This provides specific evidence for experimentally induced plasticity (tDCS) and dependent use plasticity (strength training) working together to provide an enhanced effect above resistance training alone.

While studying the modulation of reflexes can be used to probe interlimb neural activity (Burke, Dickson, & Skuse, 1991; Zehr et al., 2004), no study has directly assessed the relative contribution of cutaneous afferent pathways to an interlimb strength transfer protocol. Providing ‘enhanced’ cutaneous stimulation during unilateral contractions may alter excitability of interlimb reflex pathways and cortical circuits potentially modifying the contralateral increase in strength. Therefore, the purpose of this study was to determine the relative contribution of cutaneous afferent pathways as a mechanism of cross‐education by directly assessing if unilateral cutaneous stimulation alters ipsilateral and contralateral strength gains. It was hypothesized that providing ‘enhanced’ sensory input via electrical stimulation during resistance training would improve strength gains compared with training alone.

## 2 METHODS

### 2.1 Participants

A total of 27 neurologically intact right‐handed participants were recruited and randomly assigned to one of three experimental groups. Handedness was determined using a 10‐item version of the Waterloo Handedness Questionnaire (WHQ) which ranged from −20 to +20, where a negative score indicates left handedness and a positive score indicates right handedness. The groups included maximal voluntary training (TRAIN) (7 females; 2 males, 22.1 ± 4.2 years, 168.2 ± 9.7cm, 69.6 ± 11.0 kg, 14.8 ± 4.4 WHQ), cutaneous nerve stimulation only (STIM) (6 females; 3 males, 23.2 ± 2.8 years, 170.8 ± 12 cm, 64.5 ± 13.2 kg, 18.4 ± 2.8 WHQ), or cutaneous nerve stimulation during maximal voluntary contraction (TRAIN + STIM) (5 females; 4 males, 22.4 ± 2.8 years, 175.1 ± 10.8 cm, 70.3 ± 16.1 kg, 15.9 ± 4.0 WHQ). Protocols used in the experiments were approved by the University of Victoria Human Research Ethics Committee and performed according to the Declaration of Helsinki (1964).

### 2.2 Experimental procedures

**Figure 1:**
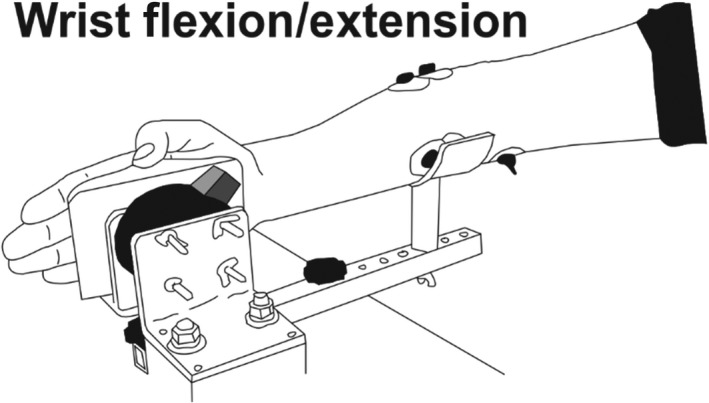
Illustration of the experimental position for measurement of peak wrist extension and flexion MVCs. EMG electrodes placed over the FCR and ECR with stimulation electrodes placed over the SR nerve at the wrist

Each participant completed two pretraining (PRE 1, PRE 2) and one posttraining (POST) session during which dependent measures of strength, muscle activation, and cutaneous reflex excitability were assessed. Multiple baseline sessions were used to account for learning effects. During these sessions, tests were performed in the same order and under the same environmental conditions (i.e., temperature, noise, lighting, and participant position) and session time of day were kept as consistent as possible as established in previous research (Dragert & Zehr, 2013; Lagerquist, Zehr, Baldwin, Klakowicz, & Collins, 2006; Zehr, 2002). Participants completed training in the right arm only within their specified group 3x/week for 5 weeks, most commonly on Monday, Wednesday, and Friday. The training program was progressive in nature, beginning with four sets of eight repetitions and increasing in volume by one additional set each training day, up to a maximum training volume of six sets of eight repetitions. The training program included a taper down to four sets of eight contractions over the final two training sessions to ensure recovery from training prior to posttest session. Each training session consisted of six sets of eight repetitions of the specified training. All training sessions were performed in a supervised laboratory setting while sitting with the right arm placed in a secured custom‐built forearm brace. For all training sessions, the forearm was secured in place with joint angles being maintained throughout training. The TRAIN group protocol consisted of unilateral maximal voluntary contractions (MVCs) of the right wrist extensors (Figure 1a). The STIM group received only cutaneous stimulation (2 times radiating threshold [RT] for 3 s at 50Hz) of the superficial radial (SR) nerve at the right wrist. Stimulation was delivered at the same relative intensity, duration, and timing across individuals and groups. This condition was chosen to provide a sham training condition as no motor response was evoked with SR stimulation. The TRAIN + STIM group protocol included MVCs of the right wrist extensors while the SR nerve was stimulated. MVCs of the wrist extensors were initiated upon sensation of the SR stimulation and released when stimulation stopped.

### 2.3 Strength – maximal voluntary contractions

MVCs of wrist extension, wrist flexion, and handgrip were assessed bilaterally at PRE1, PRE2, and POST. Three MVCs were recorded for each task bilaterally and held for 3 s each with 1 min of rest between contractions. All MVCs were recorded in a seated position with the nontested arm placed in the participant’s lap. Wrist extension and flexion were assessed in a custom‐built forearm support attached to a Gamma Sensor force transducer (ATI Industrial Automation, Model FT06598). The forearm was secured, and all joint angles were maintained across testing time points. Handgrip MVC was recorded via dynamometer in the same seated position at an approximate 45° angle away from the body. All settings were maintained through the data collection process. Verbal encouragement was provided by the tester in a similar fashion for all measures and time points. Although wrist extension was the primary strength measure, forearm strength assessments were performed in a manipulandum to assess for transfer of strength to the untrained limb across multiple tasks using the same musculature (Figure 1). For each task, the contraction with the highest peak force was used for comparison at each time point. Participants were familiarized with the isometric strength tasks prior to MVCs and completed a standardized warm‐up prior to each session.

### 2.4 Muscle activation – electromyography

Electromyography was recorded bilaterally from the muscle bellies of the flexor carpi radialis (FCR) and extensor carpi radialis (ECR) in the forearm. After the skin was cleaned with alcohol wipes, surface electrodes (Thought Technologies Ltd.) were placed in a bipolar configuration on the skin using a 2‐cm interelectrode distance, oriented along the fiber direction, in accordance with SENIAM procedures (Hermens, Freriks, Disselhorst‐Klug, & Rau, 2000). A reference electrode was placed on the medial epicondyle to serve as a common ground for the EMG signal. Electrodes were placed in the same position at each testing session. Landmarking measurements were recorded at the initial pretest to ensure correct placement at each subsequent time point. During MVCs a 0.5 s window of time around peak muscle activity was used to calculate the peak mean absolute value (MAV). The peak MAV associated with the corresponding peak MVC from each baseline and posttest measure was used for assessment.

EMG was preamplified 5000x (GRASS P511, AstroMed, Inc.) and band‐pass filtered 100–300 Hz. The output was sent to the A/D interface (National Instruments Corp. TX, USA) where it was converted into a digital signal and sampled at 1,000 Hz using custom‐built continuous acquisition software (LabVIEW, National Instruments) and stored to a computer for off‐line analysis.

### 2.5 Sensory stimulation

#### 2.5.1 Cutaneous reflexes

Cutaneous reflexes were evoked via stimulation of the superficial radial nerve (SR) innervating the dorsum of the hand. Electrodes for SR nerve stimulation were placed just proximal to the radial head (Zehr & Chua, 2000; Zehr & Duysens, 2004). Appropriate stimulation location was checked by ensuring that radiating paresthesia was evoked into the appropriate cutaneous innervation area of the SR. To assess cutaneous reflexes, trains of 5 x 1.0 ms pulses at 300 Hz were delivered at an intensity of 3 x RT via isolated constant current stimulator (Grass S88 stimulator with SIU5 stimulus isolation and a CCU1 constant current unit AstroMed‐Grass Inc., Canada). To provide the same relative intensity of stimulation between participants, a multiple of the radiating threshold (RT) was used. RT was determined as the minimum intensity that evokes a clear radiating sensation in the entire perceptive field the SR nerve innervates (Brooke et al., 1997; De Serres, Yang, & Patrick, 1995; Delwaide, Crenna, & Fleron, 1981; Duysens, Trippel, Horstmann, & Dietz, 1990). Cutaneous reflexes were assessed during graded ipsilateral contractions of the wrist extensors of 5, 10, 25, and 50% of EMG_max_. The level of background activity significantly modulates cutaneous responses such that as activity increases, the reflex response also increases in a linear fashion (Aniss et al., 1992; Burke et al., 1991; Komiyama, Zehr, & Stein, 2000; Van Wezel, Ottenhoff, & Duysens, 1997; Yang & Stein, 1990).

#### 2.5.2 Enhanced sensory stimulation

For the groups that received enhanced cutaneous stimulation during their training, trains of 1.0ms pulses at 50 Hz were delivered at an intensity of 2 x RT for 3 s (equal duration to training MVCs). Fifty Hz frequency was chosen as it most closely resembles the sensation of surface pressure on the back of the hand during wrist extension. Cutaneous stimulation intensity was set low enough to producing a buzzing or fluttering sensation in the innervation area without producing measurable changes in motor output (Zehr, 2006; Zehr & Stein, 1999). Nonnoxious stimulation intensities were found for each participant to ensure nonnociceptive pathways were stimulated.

### 2.6 Data analysis

EMG data were analyzed for background amplitudes and reflexes using custom‐written software program (MATLAB, The Mathworks, Inc.). The net effect of cutaneous input on motoneuron excitability is inferred from surface EMG recorded in the muscle of interest. Modulation of ongoing activity can be seen by averaging data that are time locked to the known stimulus. The reflex response was determined by averaging 20 sweeps of SR stimulation then subtracting the prestimulation activity, leaving reflex activity to be assessed (Brooke et al., 1997; Zehr & Stein, 1999). This technique allows for measurement of both facilitatory and inhibitory responses (Baken, Dietz, & Duysens, 2005). Monitoring the effect of cutaneous stimulation on muscle activity provides reasonable temporal resolution to accurately document the amplitude and latency of the responses (Brooke et al., 1997). The stimulus artifact was removed from the subtracted reflex trace and data were then low‐pass filtered at 30 Hz using a dual‐pass, fourth‐order Butterworth filter.

Initially, reflexes were quantified as the average cumulative reflex over 150 ms following stimulation. This value is determined as the integral obtained at 150 ms divided by the time interval of integration to yield the overall reflex effect. If the value is positive, overall facilitation has occurred; if the value is negative, overall inhibition has occurred. This quantification method allows for interpretation of modulation of reflex pathways from spinal, brainstem, and supraspinal centers (Komiyama et al., 2000). Triphasic responses at varying delay latencies, which can be excitatory or inhibitory, were recorded bilaterally in the ECR and FCR during graded ipsilateral wrist extension contractions. (De Serres et al., 1995; Duysens, Tax, Trippel, & Dietz, 1992; Gibbs, Harrison, & Stephens, 1995; Jenner & Stephens, 1982; Van Wezel et al., 1997; Yang & Stein, 1990). An early latency component was identified as occurring before 75 ms, the middle component between 70 and 120 ms, and the late component measured after 120 ms (Brooke et al., 1997; Duysens et al., 1992). The time window for each latency was visually chosen around the peak response which was said to be a significant reflex if the peak was 2 standard deviations outside of the background muscle activity (Zehr & Chua, 2000). Within each time window, all data were averaged together, and a 10 ms band around the maximum response was used to obtain a single value. All reflex measures were normalized to the corresponding maximally evoked motor response (M_max_).

### 2.7 Statistics

Using commercially available software (SPSS 20.0, Chicago, IL), strength data were analyzed using a one‐way ANCOVA with the between‐subjects factor of group, using pretest scores as the covariate and posttest scores as the dependent variable. This was performed due to baseline differences in strength between groups in both the right and left arms. Peak muscle activity, PT, and RT data were analyzed using a between‐within 3 (Group; TRAIN, STIM, TRAIN + STIM) x 2 (Time; PRE_avg_, POST) ANOVA with each muscle tested separately. Background muscle activity and cutaneous reflex data were analyzed using a between‐within 3 (Group) x 2 (Time) x 4 (Contraction intensity; 5, 10, 25, 50% EMG_max_) ANOVA. For cutaneous reflex data, a priori comparisons within each training group were also assessed by a 2 (Time) x 3 (Contraction intensity) repeated‐measures ANOVA. M_max_ data were analyzed using a between‐within 3 (Group) x 2 (Time) ANOVA with each muscle tested separately. Each arm was tested separately in analysis. M_max_ was used to normalize peak muscle activity and reflex measures at each time point. If significant main effects or interactions were detected, simple main effects analysis followed using one‐way ANOVA and LSD post hoc or pairwise comparisons where appropriate. A Cohen’s d value of effect size was determined for all significant results. Assumptions for ANOVA and paired‐samples *t* tests were evaluated for parametric tests for a within‐subject design. Statistical significance was set at *p* ≤ .05.

## 3 RESULTS

### 3.1 Strength – maximal voluntary contractions

**Figure 2:**
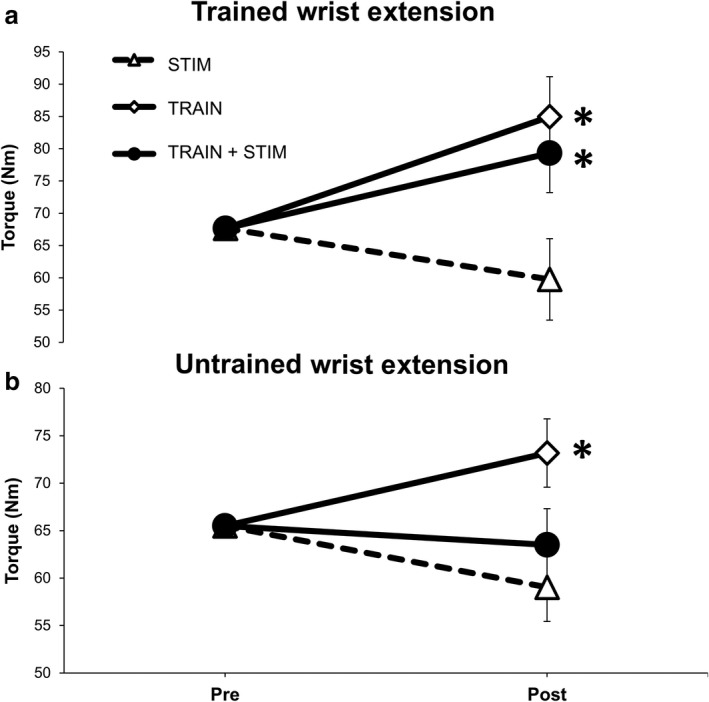
Effects of 5 weeks (15 sessions) of unilateral wrist extension training on peak wrist extension strength in the (a) trained (right); (b) untrained (left) limb. * Indicates a significant increase in strength from the adjusted premeasure score after 5 weeks of unilateral wrist extension training. Values are mean ± SE (*p* < .05)

Results indicate that 5 weeks of voluntary wrist extension training increases strength in the trained wrist extensors regardless of cutaneous stimulation. One‐way ANCOVA indicated a significant effect of GROUP (*F*_(2,23)_ = 4.809, *p* = .018). Adjusted wrist extension torque at POST was significantly higher than STIM in both the TRAIN (85.0 ± 6.2 Nm vs. 59.8 ± 6.1 Nm; *p* = .004) and TRAIN + STIM Groups (79.3 ± 6.3 Nm vs. 59.8 ± 6.1 Nm; *p* = .037) with no difference between TRAIN and TRAIN + STIM (*p* = .538) (Figure 2).

In the untrained contralateral limb, results indicate that the TRAIN group increased peak wrist extension torque after the intervention. One‐way ANCOVA indicated a significant effect of GROUP (*F*_(2,23)_ = 5.101, *p* = .015). After the intervention, adjusted wrist extension torque was significantly higher in the TRAIN group compared with STIM (73.9 ± 3.5 Nm vs. 58.8 Nm; *p* = .005) and TRAIN + STIM (73.9 ± 3.5 Nm vs. 63.0 ± 3.7 Nm; *p* = .049). Levene’s test and normality checks were carried out and the assumptions met.

There were no significant differences in peak strength after training for any group during peak handgrip and wrist flexion contractions (*p* > .05) (Table 1).

**Table 1:** Adjusted Strength measures

	PRE_ADJUSTED_	POST_TRAIN_	POST_STIM_	POST_TRAIN+STIM_	Sig.
Right Handgrip	36.1 kg	36.8 ± 2.0 kg	35.2 ± 1.9 kg	35.5 ± 2.0 kg	NS.
Left Handgrip	35.6 kg	34.2 ± 2.3 kg	34.5 ± 2.2 kg	34.7 ± 2.3 kg	NS.
Right Flexion	85.0 Nm	82.3 ± 16.8 Nm	74.2 ± 16.4 Nm	89.2 ± 18.2 Nm	NS.
Left Flexion	66.9 Nm	72.8 ± 17.8 Nm	64.9 ± 17.5 Nm	73.5 ± 18.1 Nm	NS.

Note

NS. No significant differences between any groups (*p* > .05).

Values are adjusted based on analysis or covariance.

### 3.2 Peak muscle activation

**Figure 3:**
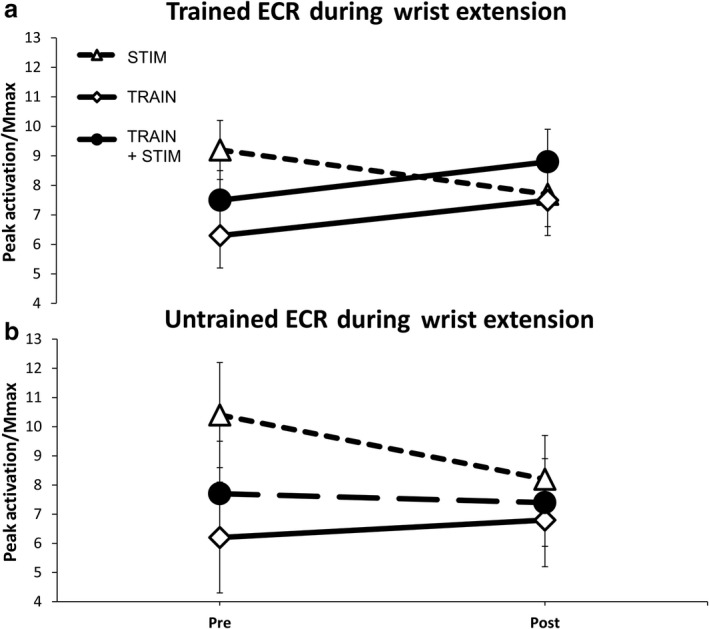
Effects of 5 weeks (15 sessions) of unilateral wrist extension training on peak muscle activation during extension MVCs in both the (a) Trained (right) and (b) Untrained (left) limb. (a) Group average of peak muscle activation in the ECR. Values are normalized to maximally evoked motor responses (M_max_). Values are mean ± SE (*p* < .05)

After 5 weeks of voluntary wrist extension training there is a differential response in peak muscle activation of the trained ECR between TRAIN and TRAIN + STIM compared with STIM only (Figure 3). Repeated‐measures ANOVA indicated a significant GROUP x TIME interaction (*F*_(2,23)_=3.816, *p* = .037). Pairwise comparisons indicate significant reduction in peak muscle activation for STIM after the intervention (9.2 ± 4.0 vs. 7.7 ± 3.4; *p* = .032). Pairwise comparisons indicate there was no significant difference in peak muscle activation after the training intervention for TRAIN or TRAIN + STIM (*p* > .05). Results of ANOVA indicate no differences in peak muscle activation of the trained ECR for handgrip or wrist flexion for any group in the untrained limb (Table 2). Results of ANOVA indicate no differences in peak muscle activation of the FCR for wrist extension, flexion, or handgrip in either limb after the intervention (*p* > .05).

**Table 2:** Peak muscle activation normalized to maximally evoked motor responses (M_max_)

Group		TRAIN		STIM		TRAIN + STIM		SIG
	Muscle	PRE	POST	PRE	POST	PRE	POST	
Right Extension	FCR	1.4 ± 1.1	1.5 ± 0.8	0.9 ± 0.6	1.2 ± 1.1	0.8 ± 0.3	1.0 ± 0.6	NS.
Left Extension	FCR	2.1 ± 1.9	1.5 ± 1.0	2.0 ± 1.8	1.5 ± 2.2	1.2 ± 1.0	1.6 ± 2.0	NS.
Right Flexion	FCR	7.6 ± 3.9	6.2 ± 3.2	5.9 ± 2.4	5.9 ± 2.4	8.7 ± 3.8	6.8 ± 3.7	NS.
	ECR	2.5 ± 1.2	1.9 ± 1.0	2.0 ± 0.9	1.8 ± 1.0	2.6 ± 1.2	1.7 ± 0.8	NS.
Left Flexion	FCR	6.5 ± 3.2	5.4 ± 2.0	7.7 ± 6.1	5.3 ± 3.1	6.4 ± 2.7	5.8 ± 3.5	NS.
	ECR	2.2 ± 0.9	1.6 ± 0.7	2.5 ± 0.9	3.0 ± 2.5	2.0 ± 1.0	2.6 ± 3.0	NS.
Right Handgrip	FCR	3.9 ± 2.5	3.5 ± 1.2	3.4 ± 2.1	3.3 ± 1.5	4.3 ± 3.8	4.0 ± 2.1	NS.
	ECR	5.9 ± 2.6	6.7 ± 3.0	8.4 ± 1.7	7.4 ± 3.3	6.9 ± 2.7	6.4 ± 5.0	NS.
Left Handgrip	FCR	4.7 ± 1.8	4.2 ± 2.0	5.1 ± 2.9	4.5 ± 2.4	4.1 ± 1.7	3.3 ± 1.9	NS.
	ECR	7.0 ± 4.0	5.4 ± 1.5	10.5 ± 5.1	11.6 ± 5.1	7.1 ± 1.7	6.0 ± 3.9	NS.

Note

NS. No significant differences between any groups (*p* > .05).

Values are normalized to maximally evoked motor responses (mV/mV*100).

Values are mean ± standard deviation.

### 3.3 Maximally evoked motor responses (M_max_)

Results indicate maximally evoked motor responses (M_max_) were similar across time points providing a valid normalization technique for EMG and reflex measures. A 3 x 3 repeated‐measures ANOVA indicated a significant effect of GROUP in the right FCR (*F*_(2,24)_=4.083, *p* = .030), left FCR (*F*_(2,24)_=5.367, *p* = .012), and left ECR (*F*_(2,24)_=3.398, *p* = .050). Group differences were expected as there were significant differences in baseline strength between groups. There was a significant main effect of TIME in the left ECR only (*F*_(2,48)_=4.057, *p* = .024). Pairwise comparisons indicate that PRE2 was significantly lower than both PRE1 (1293.6 ± 67.9 µV vs. 1388.5 ± 66.2 µV; *p* = .022) and POST (1293.6 ± 67.9 µV vs. 1395.4 ± 67.9 µV; *p* = .012). There were no significant differences in M_max_ over any time point in the right FCR, right ECR, and left ECR (*p* > .05).

### 3.4 Background EMG during cutaneous reflexes

**Figure 4:**
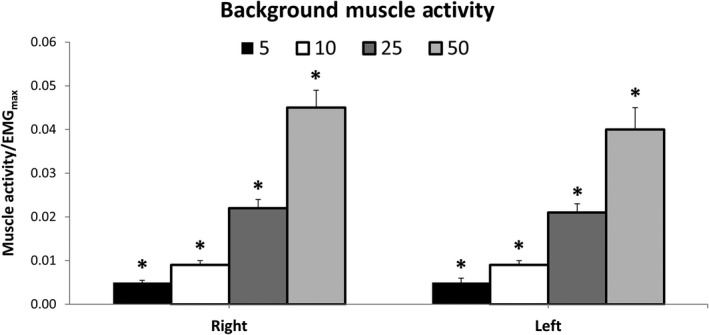
Background muscle activity during cutaneous reflex measurement pooled across group and time. Bar graphs are presented for both the trained (right) and untrained (left) arms at contraction intensities of 5, 10, 25, and 50% EMG_max_. Values are normalized to EMG during maximal voluntary contraction (EMG_max_). * Indicates significant differences between all other contraction intensities

Results from the 3 x 2 x 4 ANOVA indicate there were no significant interactions or main effects of group or time for background muscle activity during cutaneous reflex measurement (*p* > .05) (Figure 4). There was a significant effect of contraction intensity in both the right (*F*_(3,72)_=96.724, *p* < .001) and left ECR (*F*_(3,72)_=76.194, *p* < .001). Pairwise comparisons indicate that there was a significant increase in muscle activity between all levels of contraction in both the right and left ECR (*p* < .001).

### 3.5 Cutaneous reflexes

Results from the 3 x 2 x 4 ANOVA assessing ACRE 150, early latency, and middle latency reflexes indicate there were no significant interactions or main effects for GROUP or TIME (*p* > .05). For ACRE 150 there was a significant main effect of contraction intensity in both the trained right (*F*_(3,72)_=28.380, *p* < .001) and untrained left limb (*F*_(3,72)_=21.494, *p* < .001). For early latency reflexes there was a significant main effect of contraction intensity in both the trained right ((*F*_(3,72)_=78.231, *p* < .001) and untrained left limb (*F*_(3,72)_=53.081, *p* < .001).

**Figure 5:**
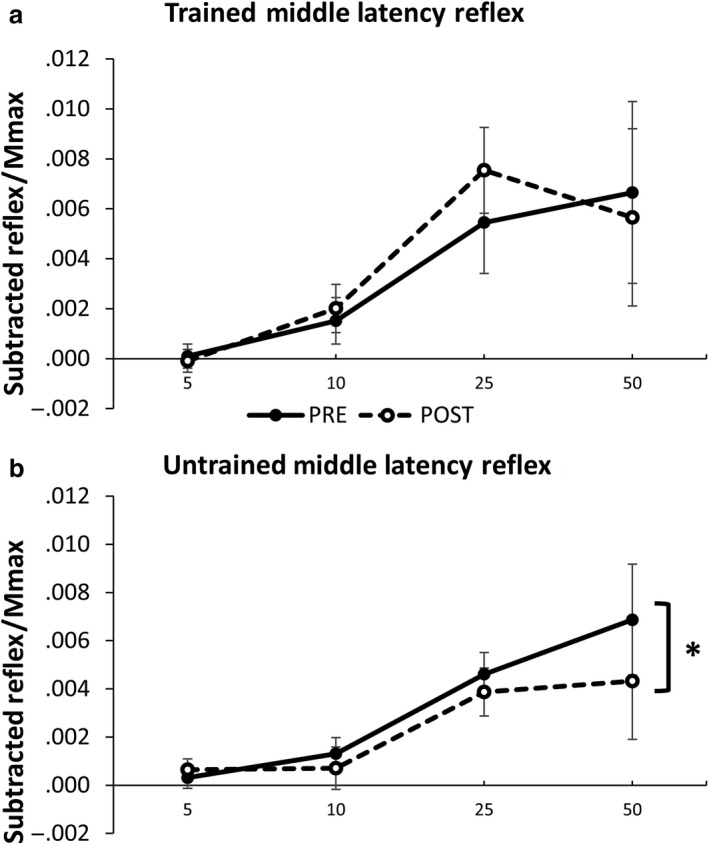
Middle latency subtracted reflex amplitude pooled across group in both the (a) Trained and (b) Untrained limb. EMG is recorded from the ECR during SR nerve stimulation. Values are displayed across contraction intensity (5, 10, 25, and 50% EMG_max_) and between pre‐ and postmeasurements. Values are normalized to maximally evoked motor responses (M_max_). * Significant main effect of time pooled across group and contraction intensity. No differences between groups were present existed. Values are mean ± SE (*p* < .05)

For middle latency in the untrained left limb there was a main effect of time pooled across contraction intensity and group (*F*_(1,24)_=6.550, *p* = .001). Results from a priori 2 x 3 ANOVAs for each group indicate no significant interactions or main effects of time for any group for either limb (*p* > .05). There was a significant main effect of contraction intensity in both the trained right ((*F*_(3,72)_=5.129, *p* = .003) and untrained left limb (*F*_(3,72)_=6.427, *p* = .001) (Figure 5).

**Figure 6:**
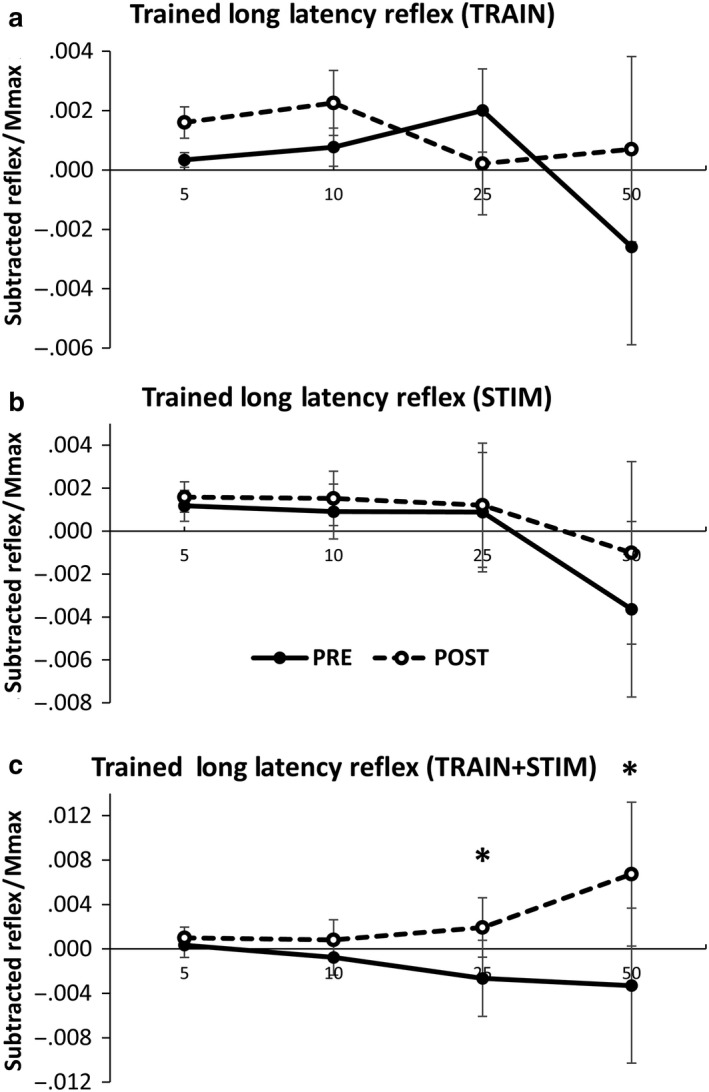
Long latency subtracted reflex amplitude in the trained limb for the (a) TRAIN, (b) STIM, and (c) TRAIN + STIM Groups. EMG is displayed for the ECR during SR nerve stimulation. Values are displayed across contraction intensity (5, 10, 25, and 50% EMG_max_) and between pre‐ and postmeasurements. Values are normalized to maximally evoked motor responses (M_max_). * Significant difference in subtracted reflex amplitude between pre‐ and post–time points in the TRAIN + STIM group only. Values are mean ± SE (*p* < .05)

**Figure 7:**
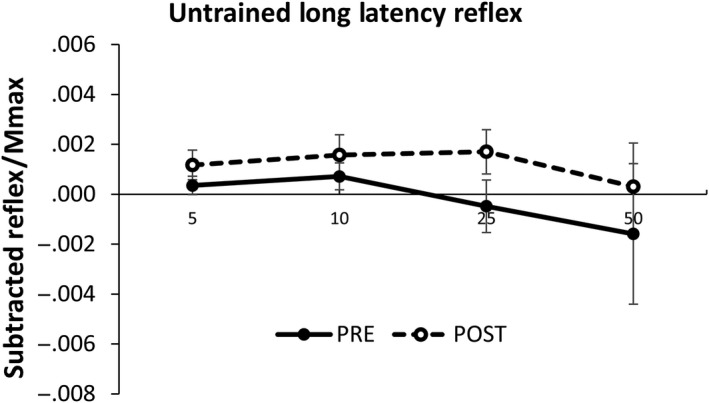
Long latency subtracted reflex amplitude in the untrained limb pooled across group. EMG is recorded from the ECR during SR nerve stimulation. Values are displayed across contraction intensity (5, 10, 25, and 50% EMG_max_) and between pre‐ and postmeasurements. Values are normalized to maximally evoked motor responses (M_max_). Values are mean ± SE (*p* < .05)

For long latency reflexes in the trained right limb there was a significant Contraction x Time Interaction (*F*_(3,24)_=4.487, *p* = .006). Results from a priori 2 x 3 ANOVAs for each group indicate no significant interactions or main effects of time for TRAIN or STIM (*p* > .05). However, for the TRAIN + STIM group there was a significant Contraction x Time Interaction (*F*_(3,24)_=8.574, *p* < .001) and time main effect (*F*_(1,8)_=14.201, *p* = .005). Paired samples *t* tests indicate a significant difference in reflex amplitude at 25% (*p* = .010) and 50% (*p* = .006) contraction intensities (Figures 6 and 7 ).

### 3.6 Perceptual and radiating thresholds

Results of the 2 x 3 ANOVA indicate that there was no change over time or across group for either perceptual or radiating threshold as no significant interactions or main effects were present (*p* > .05) (Table 3.

**Table 3:** Perceptual and radiating thresholds

	PRE_avg_ (mA)	POST (mA)	Significance
Right PT	1.6 ± 0.37	1.6 ± 0.5	NS.
Right RT	4.5 ± 0.6	4.9 ± 1.3	NS.
Left PT	1.7 ± 0.4	1.6 ± 0.4	NS.
Left RT	4.3 ± 0.7	4.5 ± 1.3	NS.

Note

NS. No significant differences between PRE/POST (*p* > .05).

Values are mean ± standard deviation.

## 4 DISCUSSION

Unilateral wrist extension training alone (TRAIN) increased peak force output in the trained wrist extensors. Providing ‘enhanced’ cutaneous sensory input via electrical stimulation during training (TRAIN + STIM) led to a similar increase in strength in the trained limb compared with TRAIN. However, the major finding of the current investigation is that providing ‘enhanced’ cutaneous input in the TRAIN + STIM group blocked interlimb strength transfer to the untrained wrist extensors. This is the first study to directly assess the cutaneous sensory contribution to interlimb strength transfer from unilateral resistance training. While it was hypothesized that providing ‘enhanced’ cutaneous input would facilitate the strength gain in the untrained contralateral side, it appears the large sensory volley may have interfered with the integration of appropriate sensorimotor cues required to facilitate an interlimb transfer and improvements in the untrained limb.

### 4.1 Absence of interlimb strength transfer with ‘enhanced’ cutaneous input

The group that received the 50 Hz enhanced cutaneous sensory stimulation during the maximal wrist extension training protocol saw no transfer of strength to the untrained limb. While this result is contrary to our hypothesis, this study highlights the important role of cutaneous afferent feedback during movement tasks.

It remains likely the timing and amplitude properties of the enhanced sensory volley which was not linked to the actual intention to contract interfered with the integration of sensory cues required to transfer strength to the untrained limb. If we consider the human nervous system to exhibit properties of a “Hebbian” synapse with “neurons firing together and wiring together”, providing a mismatched sensory volley may have altered fundamental properties associated with acquisition of novel motor skills (Carson, 2006; Cooper & Donald, 2005). Neurons that release action potentials at the same time have an increased probability of forming synaptic connections, while uncorrelated activity diminishes functional connectivity (Russmann et al., 2009). In typical motor behavior sensorimotor integration is tightly related to motor output and anticipated sensory afference. The compelling observation here that mistimed sensory input could completely abolish a robust neural transfer effect argues strongly for future work exploring timing.

### 4.2 Possible cortical interactions with ‘enhanced’ cutaneous feedback

Providing a large and asynchronous sensory volley during unilateral resistance training may interact with many of the same cortical areas which contribute to cross‐education. Changes in multiple cortical areas in both hemispheres have been shown after unilateral training via fMRI (Farthing, Borowsky, Chilibeck, Binsted, & Sarty, 2007; Farthing et al., 2011), positron emission tomography (PET) (Dettmers et al., 1995), and TMS (Boroojerdi, Ziemann, Chen, Butefisch, & Cohen, 2001; Hortobágyi et al., 2003; Kristeva, Cheyne, & Deecke, 1991; Perez et al., 2007). As well, adaptations in connections between primary motor cortices (M1) through transcallosal routes have shown significant plasticity with training (Hortobagyi et al., 2011; Perez et al., 2007). Plasticity of interhemispheric connections mediating cross‐education of a simple motor task likely also contribute to such effects of transfer (Hortobágyi et al., 2011). While the current investigation cannot provide insight into areas of possible integration, it is likely that interference with the cortical mechanisms of adaptation interfered with the transfer of strength to the untrained limb.

While this study is unique in that enhanced cutaneous input appears to ‘block’ the interlimb transfer of strength, previous investigators have shown an ability to alter transfer of skill or strength which may provide information regarding possible sites of interference within the current investigation. Unilateral practice of a ballistic finger abduction task has been shown to improve performance by 82% in the untrained left hand and was accompanied by bilateral increases in corticospinal excitability (Carroll, Lee, Hsu, & Sayde, 2008). A follow‐up paper found bilateral increases in performance and corticospinal excitability after unilateral training of a ballistic motor task (Lee, Kilbreath, Singh, Zeman, & Davis, 2010). Most interestingly, repetitive TMS was applied to the trained and untrained motor cortex to induce a ‘virtual lesion’. This was induced by applying repeated TMS (rTMS) to either the right or left cortex, which reduced performance gains in the contralateral hand. Researchers concluded that early retention of ballistic performance improvements in the untrained limb is due to adaptations in the untrained motor cortex.

The ability to alter the acquisition of a novel motor task through interhemispheric excitability of both sensory and motor areas of the cortex has been shown using a diverse array of tasks. After completing a single unilateral exercise session of pinch grip, participants improved their error of force in the untrained contralateral hand by almost one third. However, when rTMS was applied to the contralateral cortex during the exercise session, no transfer of improved error of force occurred (Goodall et al., 2013). Local tonic cutaneous pain induced by capsaicin cream also interferes with the retention of a newly learned locomotor adaptation task despite the finding that baseline gait and motor acquisition were unimpaired by pain (Bouffard, Bouyer, Roy, & Mercier, 2014). Taken together, these studies indicate that cutaneous sensory information can have dramatic effects on the retention of a novel motor task, and interference of interhemispheric connections may be a contributing site of adaptation. Interestingly, providing a combination of anodal tDCS over M1 during a single session increased force production in the untrained limb, while training with sham tDCS or anodal tDCS alone showed no increase in contralateral strength (Hendy & Kidgell, 2014). This was accompanied with changes in interhemispheric inhibition and corticospinal excitability in the untrained limb in the group that received anodal tDCS. It becomes apparent that learning, regardless of the type or task, can be transferred between hemispheres and directly impacted by altering excitability of the cortex facilitating this transmission.

The main result from the current investigation of a ‘block’ of transfer between limbs may be due to the unilateral training task being paired with a mismatched sensory volley not allowing for appropriate integration of sensory cues for transfer. This is similar to recent work where ‘enhanced’ somatosensory input via prolonged low‐amplitude somatosensory electric stimulation (SES) with nerve stimulation was provided. SES stimulation alone has been shown to facilitate transfer of performance of visuomotor task to the untrained contralateral limb (Veldman et al., 2014). However, while SES applied to the median and radial nerves alone as well as visuomotor task training improve performance in both the trained and untrained limbs, little to no additional benefit appears to be provided by applying low‐amplitude nerve stimulation during the visuomotor training task (Négyesi et al., 2018; Veldman et al., 2015).

### 4.3 Possible spinal mechanisms with enhanced cutaneous feedback

Here, cutaneous reflexes provided a measure of whether the integration of sensory information from the skin was differentially relayed after resistance training, electrical stimulation, or a combination of both. Once cutaneous mechanoreceptors are activated, sensory information diverges through an unknown number of polysynaptic connections and is integrated at multiple levels of the nervous system, and subsequently modulates ongoing muscle activity (Zehr, 2006; Zehr & Stein, 1999). Within the current investigation there was a significant facilitation of long latency reflex amplitude in the trained limb after the intervention for the TRAIN + STIM group. This demonstrates interaction between the ascending afferent pathways and the sensorimotor connections, facilitating reflex transition after resistance training in the trained limb. This is the first evidence of altered transmission of cutaneous afferent information with resistance training (Figure 6c). The multicomponent EMG response to cutaneous nerve stimulation is thought to arise due to differences in the number of interneurons in a particular pathway within the spinal cord (Zehr, 2006; Zehr & Stein, 1999). Based on the latency of the earliest responses, it is assumed that the earliest components of cutaneous reflexes can be mediated by pathways in the spinal cord (Baken et al., 2005; Dimitrijevic & Nathan, 1969; Zehr & Stein, 1999). Responses at longer latencies are likely the result of transmission through longer pathways which may contain multiple interneurons at multiple levels of the nervous system including cortical contributions (Eccles & Lundberg, 1959; Jenner & Stephens, 1982; Nielsen, Petersen, & Fedirchuk, 1997). As the facilitation was only seen in the group that received cutaneous stimulation during voluntary training, there appears to be an interaction between the two conditions which led to a long‐term alteration in excitability. This change in excitability may be related to the lack of strength transfer to the untrained cortex within this group as there were no changes in early and middle latency reflex excitability within this or any group.

While there are no direct connections between motoneurons on the contralateral side, afferents do modulate interlimb coordination (Sherrington, 1910) and are most likely mediated through commissural interneurons (Jankowska, Krutki, & Matsuyama, 2005) and propriospinal paths (Burke, Gracies, Mazevet, Meunier, & Pierrot‐Deseilligny, 1992; Jankowska, 2001). Activation of group 1a afferents inhibit contralateral homologous motoneurons (McCrea, 2001) via the Ia inhibitory interneurons (Delwaide & Pepin, 1991). This has been functionally demonstrated as contraction of an ipsilateral limb has been shown to depress H‐reflex amplitude in the homologous contralateral muscle (Carson et al., 2004; Hortobágyi et al., 2003).

There was no change over time and between any of the groups for early latency subtracted reflex amplitude indicating effects are unlikely to be occurring purely in the spinal cord. The current investigation indicates excitability of cutaneous spinal reflex pathways is not altered with unilateral resistance training or repeated sensory volleys evoked with electrical stimulation. Other studies that have found alterations in spinal reflex excitability have done so using techniques such as the H‐reflex (Dragert & Zehr, 2011; Lagerquist, Zehr, Baldwin, et al., 2006) or reciprocal inhibition (Dragert & Zehr, 2013; Geertsen et al., 2008) which assess predominantly muscle afferents.

### 4.4 Possible measures of altered cutaneous transmission

We thought that receiving cutaneous stimulation at a reasonably strong intensity over a period of 5 weeks may induce some type of chronic adaptation in detection thresholds of cutaneous afferents (Volkmann, 1858). If so, it could be detected by measuring either the PT or RT of the SR nerve at the wrist before and after the intervention. However, after 5 weeks of electrical nerve stimulation applied to the superficial radial cutaneous nerve, there were no differences for any group or time point for PT or RT indicating little adaptation to the detection thresholds or excitability of transmission with repeated activation or resistance training.

### 4.5 Controls within the current investigation

A type of sham condition was used as the control group to test whether repeated cutaneous stimulation alone would provide alterations in excitability or strength changes in the trained or untrained limb. Participants who were assigned to the STIM group only received passive SR stimulation which did not produce any motor response. This passive stimulation was the same volume and in the same position as the groups completing voluntary contractions. The participants who only received STIM did not increase in strength, muscle activation, or reflex excitability, indicating that cutaneous stimulation by itself had little to no impact on motor output during voluntary contractions.

### 4.6 Limitations and future directions

A limitation in the current investigation was the timing and intensity of electrical stimulation used to ‘enhance’ sensory input. In the TRAIN + STIM group, each maximal wrist extension contraction throughout the training protocol was initiated by the electrical stimulator, therefore, a volley of cutaneous sensory information was provided prior to initiation of the contraction. In contrast, initiating the movement produces the appropriate volley of sensory information based on timing, intensity, and task. Not only was the timing of the stimulation provided mismatched but also the intensity of the stimulation remained constant throughout each contraction for the duration of the study. A more natural type of stimulation would have been to increase the stimulation frequency proportionally as muscle activation increased. As well, the intensity of the sensory volley may have been too high, bombarding sensorimotor cortical areas (Blickenstorfer et al., 2009; Han et al., 2003). This may not have allowed appropriate sensory cues from the wrist extension contractions to be incorporated and shared between hemispheres (Ruddy & Carson, 2013). It remains possible that an appropriately timed stimulus that is more natural and with intensity‐dependent frequency and amplitude may have shown different results under the same experimental settings.

Another limitation of the current investigation was the inability to assess the effect cutaneous stimulation to the SR nerve during wrist extension contractions had on the peak force production within each training session. It is possible that the voluntary drive during each training session was altered due to the large sensory volley. However, the similar improvement in strength between the TRAIN + STIM and TRAIN in the trained limb indicates a similar level of effectiveness of the training intervention.

Future research should explore how ‘enhanced’ cutaneous sensory information would have impacted strength transfer if applied to the untrained arm during unilateral training. Results from a recent cross‐sectional investigation indicate that enhanced stimulation on the nondominant arm amplifies interneuronal excitability in interlimb cutaneous pathways during a static task (Sun & Zehr, 2019). While this was initially part of the study design, due to constraints on the number of conditions and comparisons it was determined to be beyond the scope of the current investigation. Taking into consideration the results from the current investigation, this may be a valuable approach which could provide the ‘enhancement’ of strength in the untrained limb we initially hypothesized. The results of the current investigation provide a clear example of the specific nature of cutaneous sensory information and the necessity to provide functionally meaningful information to the nervous system.

## 5 CONCLUSIONS

Providing ‘enhanced’ sensory input via electrical stimulation during training (TRAIN + STIM) led to similar increases in strength in the trained limb compared with TRAIN. However, providing a large sensory volley during training in the TRAIN + STIM group alleviated any interlimb strength transfer to the untrained wrist extensors. It appears that the large mismatched sensory volley may have interfered with the integration of appropriate sensorimotor cues required to facilitate improvement in the untrained limb. Voluntary wrist extension training or repeated electrical stimulation to a cutaneous nerve does not appear to alter cutaneous reflex transmission across contraction intensity or latencies of response. However, receiving a large sensory volley during wrist extension training altered long‐latency cutaneous reflex amplitude from inhibition to facilitation at high levels of muscle contraction on the trained right side. While it appears that stimulation delivered under the current conditions without specific timing will not facilitate cross‐education, this provides important insight into the important contribution of appropriate cutaneous information on motor output.

## CONFLICT OF INTEREST

None of the authors have potential conflicts of interest to be disclosed.
